# Tigers, Terrain, and Human Settlement Influence the Occupancy of Leopards (*Panthera pardus*) in Southwestern Tarai, Nepal

**DOI:** 10.1002/ece3.70898

**Published:** 2025-01-20

**Authors:** Laxmi Raj Joshi, Rabin Bahadur K. C., Madhu Chetri, Morten Odden, Olivier Devineau, Ajay Karki, Bhagawan Raj Dahal, Naresh Subedi

**Affiliations:** ^1^ National Trust for Nature Conservation Lalitpur Nepal; ^2^ Faculty of Applied Ecology, Agricultural Sciences and Biotechnology Inland Norway University of Applied Sciences Koppang Norway; ^3^ Department of National Parks and Wildlife Conservation Kathmandu Nepal; ^4^ Department of Zoology and Physiology, Haub School of Environment and Natural Resources University of Wyoming Laramie Wyoming USA; ^5^ Zoological Society of London Nepal Office Kathmandu Nepal

**Keywords:** camera trap, detection, leopard occupancy, *Panthera tigris*, Shuklaphanta national park

## Abstract

Maintaining a healthy population of common leopards, a highly adaptive felid, requires updated information on their spatial occurrence. In Nepal's Tarai region, leopards coexist with tigers, which are well‐studied felid throughout its range. However, knowledge is very scarce on the patterns of leopard occupancy. We conducted an occupancy survey using remote cameras in southwestern Tarai, particularly in Shuklaphanta National Park, Nepal, to assess habitat use by leopards from December 2022 to January 2023. Naive and model‐averaged occupancy estimates were 0.51 and 0.6563 (SE: 0.022, 95% CI: 0.612, 0.70), respectively. The detection of leopards was negatively correlated with the presence of tigers. Leopard occupancy was higher closer to human settlement and higher in rugged terrain. At a time when Nepal has achieved its tiger conservation targets, efforts are required to maintain adequate prey biomass to minimize fatal encounters between tigers and leopards and displacement of leopards peripheral to the settlement area, where villagers might kill them in retaliation of livestock killing. Long‐term monitoring is required to improve understanding of the interaction between leopards, tigers, and humans in the Tarai region of Nepal.

## Graphical abstract

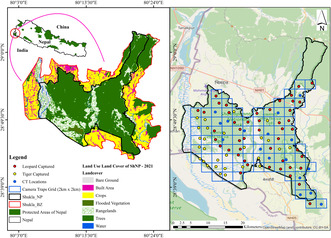

## 1 Introduction

The common leopard ( *Panthera pardus* , “leopard” hereafter) is a widely distributed felid with a broad range of prey, behavioral adaptations, and habitats (Jacobson et al. 2016; Mizutani and Jewell 1998; Nowell and Jackson 1996; Odden et al. 2014). They have developed a high resilience to human activities and a great ability to sustain themselves in highly modified and fragmented landscapes (Havmøller et al. 2019; Müller et al. 2022) as well as in a periphery to the settlement area (Athreya et al. 2013; Kafley et al. 2019; Odden, Wegge, and Fredriksen 2010). However, the species currently occupies only about 25%–37% of its historic range, and approximately 17% of the leopard range is under protection (Jacobson et al. 2016). Leopards are listed as “Vulnerable” in the IUCN Red List of Threatened Species (Stein et al. 2024). The most important causes of the global decline of leopard populations are poaching and illegal trade of body parts (Packer et al. 2011), and deliberate killing (Swanepoel et al. 2013), often resulting from increasing conflicts with humans (Baral et al. 2022).

In several protected areas in Asia, leopards compete with tigers ( *Panthera tigris tigris* ) (Barber‐Meyer et al. 2013; Jacobson et al. 2016). Spatial segregation between these two large predators has been well examined (Carter et al. 2015; Kafley et al. 2019; Thapa et al. 2021). Some studies have reported intraguild competition (Kafley et al. 2019), in which the dominant predator's distribution matches its resources, whereas intermediate predators make tradeoffs between food and safety (Steinmetz, Seuaturien, and Chutipong 2013). However, leopards do not necessarily show spatial avoidance of the areas highly used by the dominant tigers (Li et al. 2019), but some studies suggest that interference competition by tigers tends to push leopards toward forest fringes and human settlements (Odden, Wegge, and Fredriksen 2010; Rayan and Linkie 2016). Ecological segregation in which the predators selectively kill different types of prey has also been observed (Karanth and Sunquist 2000).

In Nepal, leopards are widely distributed throughout various geographical regions. Recently, leopards were also observed in snow leopard habitat ( *Panthera uncia* ) at an elevation of 4260 m in Nepal (Chetri et al. 2023). However, factors affecting leopard distribution is poorly understood (Lamichhane et al. 2021). It is important that conservation initiatives targeting the recovery of predator guilds carefully examine interspecific interactions (Harihar, Pandav, and Goyal 2011). Also, understanding how large carnivore guilds survive in human‐dominated landscapes is a key to devise conservation strategies in the face of global carnivore declines (Li et al. 2019).

At a time when Nepal has shown remarkable achievement in securing a growing tiger population, questions are being raised about the future of leopards. It is generally believed that with the increasing number of tigers, leopard may be pushed toward the marginal habitats near the buffer zones of the protected areas (Kafley et al. 2019; Lamichhane et al. 2021). However, this competitive relationship between tigers and leopards is not fully understood when considering small, protected areas like Shuklaphanta National Park (ShNP), which are surrounded by human settlements. A careful examination of the roles of influencing covariates is essential to understand the ecological niche and requirements of globally vulnerable leopards.

In the present study, we report the occupancy of leopards using remote camera data. We hypothesized that (i) leopards, as the subordinate competitor, would avoid tigers spatially to reduce the risk of potentially fatal encounters and (ii) proximity to humans and livestock would positively influence leopard occupancy. The findings are very important to devise leopard conservation priorities in the human‐dominated western Tarai landscape of Nepal.

## 2 Materials and Methods

### 2.1 Study Area

**FIGURE 1:**
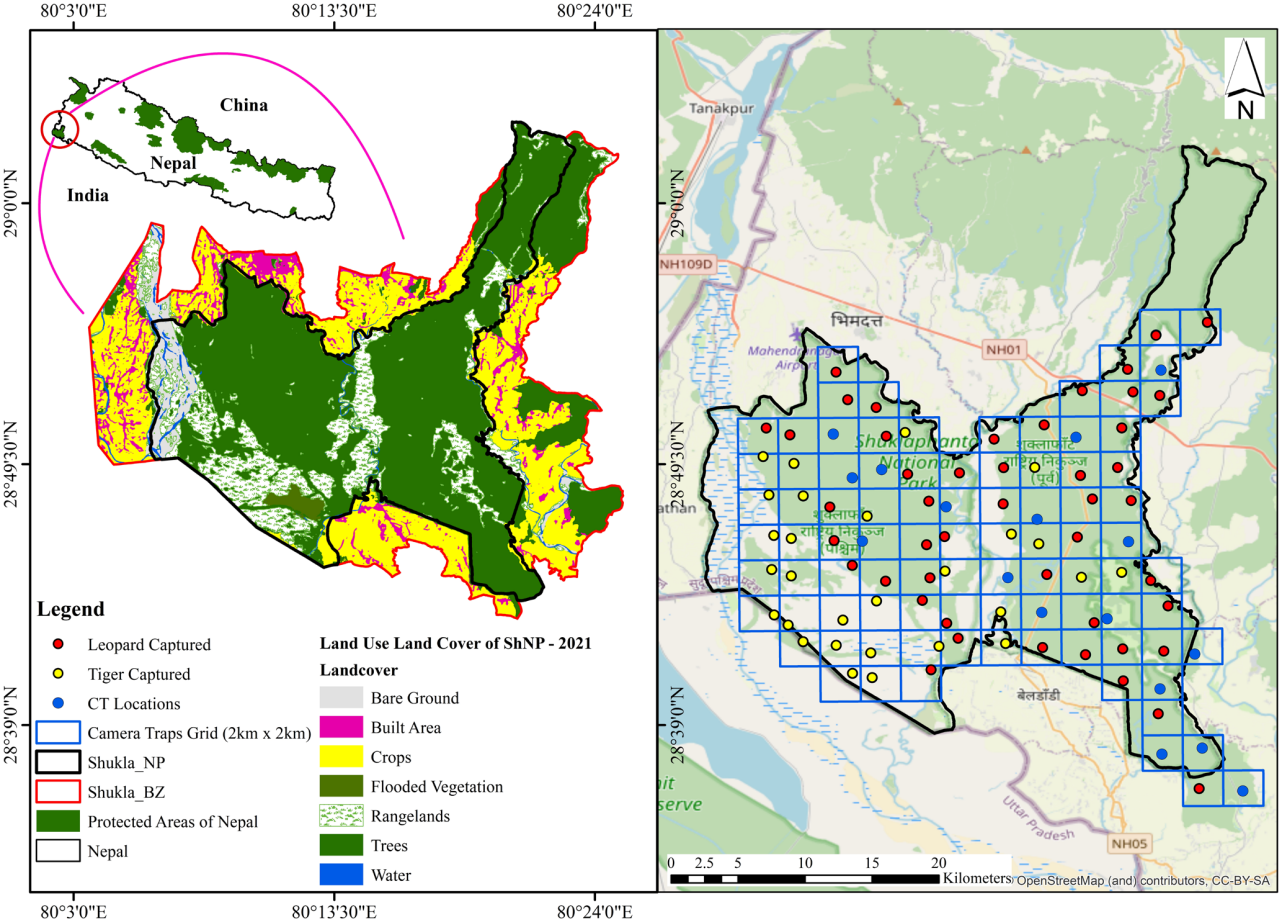
Map of the study area and camera trap layout (survey grid cells = 4 sq. km.), with locations of photographed tigers and leopards in Shuklaphanta National Park, Nepal.

The study was carried out in ShNP (28°45′47′′–29°02′52′′N and 80°05′45′′–80°21′4′′E; 175–1300 masl) of Nepal (Figure 1). ShNP lies in the southwestern Tarai and Siwalik regions of Nepal, with a core area of 305 km^2^ and 245 km^2^ of buffer zone (ShNP 2022). Established in 1976 as a Wildlife Reserve to conserve the largest population of swamp deer (*Recervus duvaucelii*) in Nepal, the park now supports a growing population of tigers and other endangered species. The transboundary movements of tigers, rhinos *(Rhinoceros unicornis)*, and elephants *(Elephas maximus)* have been well‐documented (Chanchani et al. 2014; Kumar Talukdar and Sinha 2013; Pradhan, Williams, and Dhakal 2011). The park serves as an important habitat for other endangered and vulnerable ungulates, such as hog deer ( *Axis porcinus* ) and sambar (*Rusa unicolar*) (ShNP 2022).

ShNP and the surrounding areas have a subtropical monsoon climate, with a mean annual rainfall of 1579 mm, most of which falls from June to September. The average monthly minimum and maximum temperatures is 7°C and 37°C, respectively. ShNP inhabits 665 plant species from 438 genera and 118 families, representing the highest diversity documented among protected areas in the Tarai region of Nepal (Poudyal et al. 2020; ShNP 2022). The park is also rich in faunal diversity and supports 56 mammalian species and 71 species of herpetofauna (Poudyal et al. 2020; Rawat et al. 2020).

### 2.2 Data Collection

A comprehensive tiger and prey base monitoring was conducted during December 2022 and January 2023 in the entire 305 km^2^ of ShNP based on the tiger monitoring protocol of Nepal 2017 (DNPWC 2017). The survey employed a total of 92 survey grid cells (see Figure 1), each measuring 4 km^2^ (2 × 2 km). *Panthera* V5 and V6 automated cameras were systematically placed in pairs in strategic locations, preferably on dirt roads, fire lines, dry riverbeds, and animal trails to maximize the detectability of tiger (Karanth et al. 2011). The camera traps were positioned between 45 and 60 cm above the ground, and they were deployed for 15–19 nights in each of the grid cells. Each camera trap was set to take three pictures per trigger with no delay (FAP mode) using white flash. These cameras were checked every morning to observe and document any tigers, leopards, or other species captured during the previous night. The survey was targeted for the tiger monitoring but as they used a similar path, we extracted the data of leopard from the same camera locations.

### 2.3 Covariate Selection

We selected a combination of seven remote‐sensing and ground‐based variables reflecting the characteristics of the landscape, habitat conditions, and anthropogenic pressure. In addition to the presence of tigers and prey, other factors, such as livestock presence, distance to settlements, distance to water sources (river), and terrain ruggedness (Appendix S1) may have important roles in shaping leopard occupancy (Kandel, Lamichhane, and Subedi 2020; Lovari et al. 2015; Odden, Wegge, and Fredriksen 2010; Odden and Wegge 2005). We extracted landscape‐level covariates, including distance to human settlement (settlement), distance to the nearest river (river) calculated using the near tool from ArcGIS 10.5, after downloading the respective river layer (https://rds.icimod.org/home/datadetail?metadataid=852) and settlement layer (https://www.openstreetmap.org/relation/184633#map=7/28.417/84.129) from International Centre for Integrated Mountain Development (ICIMOD) and Open Street Map (OSM). Similarly, the terrain ruggedness index (tri) for each grid cell calculated using the Shuttle Radar Topographic Mission (SRTM) Digital Elevation Model data at 90‐m resolution (downloaded from https://srtm.csi.cgiar.org/).

The presence of tigers in each grid cell was determined by the number of photographs of individual tigers captured in that cell. Leopard occupancy is likely influenced by the availability of wild prey, so an index of wild prey presence was created by summing all photographs of potential prey species (spotted deer, swamp deer, hog deer, barking deer, sambar, nilgai, and wild boar) within each grid cell. Similarly, livestock presence, which can both provide food (positive effect) and lead to conflict and retaliation (negative effect), was calculated based on the number of photographs of livestock species (goat, cow, oxen, and buffalo) in each grid cell.

To standardize the data, the capture rate for tigers, prey species, and livestock in each grid cell was calculated as the number of photo events per 100 trap nights (number of photo events/100 trap nights). This provides a consistent measure of the detection frequency for each species in relation to the total camera trap effort. However, to be included in the analysis, a species must have a minimum of 15 photo events, ensuring that the data are reliable and statistically meaningful for the analysis. All covariates were first checked for collinearity. The results showed that none of the covariates were significantly correlated (Pearson's |*r*| = < 0.5). We scaled all covariates before running the occupancy models (Krishna, Krishnaswamy, and Kumar 2008; Panthi et al. 2017).

### 2.4 Statistical Analysis

We used a single‐species, single‐season occupancy model to analyze the habitat use of leopards because it accounts for imperfect detection and uses presence–absence data for robust estimates of probability of use (Ψ) (Mackenzie et al. 2002; Mackenzie 2006). We used an extension of the standard occupancy model (Mackenzie et al. 2002), which allows the use of spatially correlated replicates (Hines et al. 2010), and analyses were carried out using with the package “unmarked” (Fiske and Chandler 2011; Kellner et al. 2023) in R (R Core Team, 2023). From the 4‐km^2^ grids, the presence or absence of leopards was recorded denoting 1 as detection and 0 as non‐detection. For the purpose of this analysis, one sampling occasion was 1 day. The grid size was selected to be greater than the mean daily movement of leopards, so that each grid could be treated as an independent sampling unit to estimate the probability of habitat‐use and such that multiple cells would constitute the home range of the species (Boyce 2006; Kshettry, Vaidyanathan, and Athreya 2017; Lele et al. 2013; Odden et al. 2014). Thus, a smaller grid size would capture the variability in space‐use within a home range or in the habitat use patterns (Srivathsa et al. 2014; Sunarto et al. 2012). We employed a two‐step modeling approach. First, we began by modeling covariates on detection probability “*p*,” where the parameter was either assumed to be constant or allowed to vary as covariates while ψ was held constant in a general model (Mackenzie 2006). Following this, site use probability (ψ) was modeled by fixing the previously identified best detection model and varying all possible combinations of site use covariates. Continuous covariates were standardized on a z‐scale, and all covariates were tested for collinearity using Pearson's correlation test and not included in the same model if *r* > 0.6 (Green 1979). Models were ranked based on their Akaike information criterion (AIC), adjusted for small sample sizes (AICc; Mackenzie 2006), and were considered to be strongly supported if they had a ΔAICc of < 2. Models that did not reach numerical convergence were excluded and not considered.

The final estimates of site use probability and detectability were calculated by model averaging the competing models (Burnham and Anderson 2002). We computed β estimates of the covariates to understand the magnitude and direction (positive or negative) of their influence on the site use and detection probability.

## 3 Results

A total of 1680 trap nights were conducted at 92 stations, resulting in a leopard detection at 47 stations and a naive occupancy estimate of 0.51. We fitted 29 (13 detection and 16 occupancy) a priori alternative model combinations of covariates to estimate leopard occupancy and detection. The model‐averaged occupancy estimate for leopards was 0.6563 (SE: 0.022, 95% CI: 0.612, 0.70).

### 3.1 Modeling Probability of Detection

**FIGURE 2:**
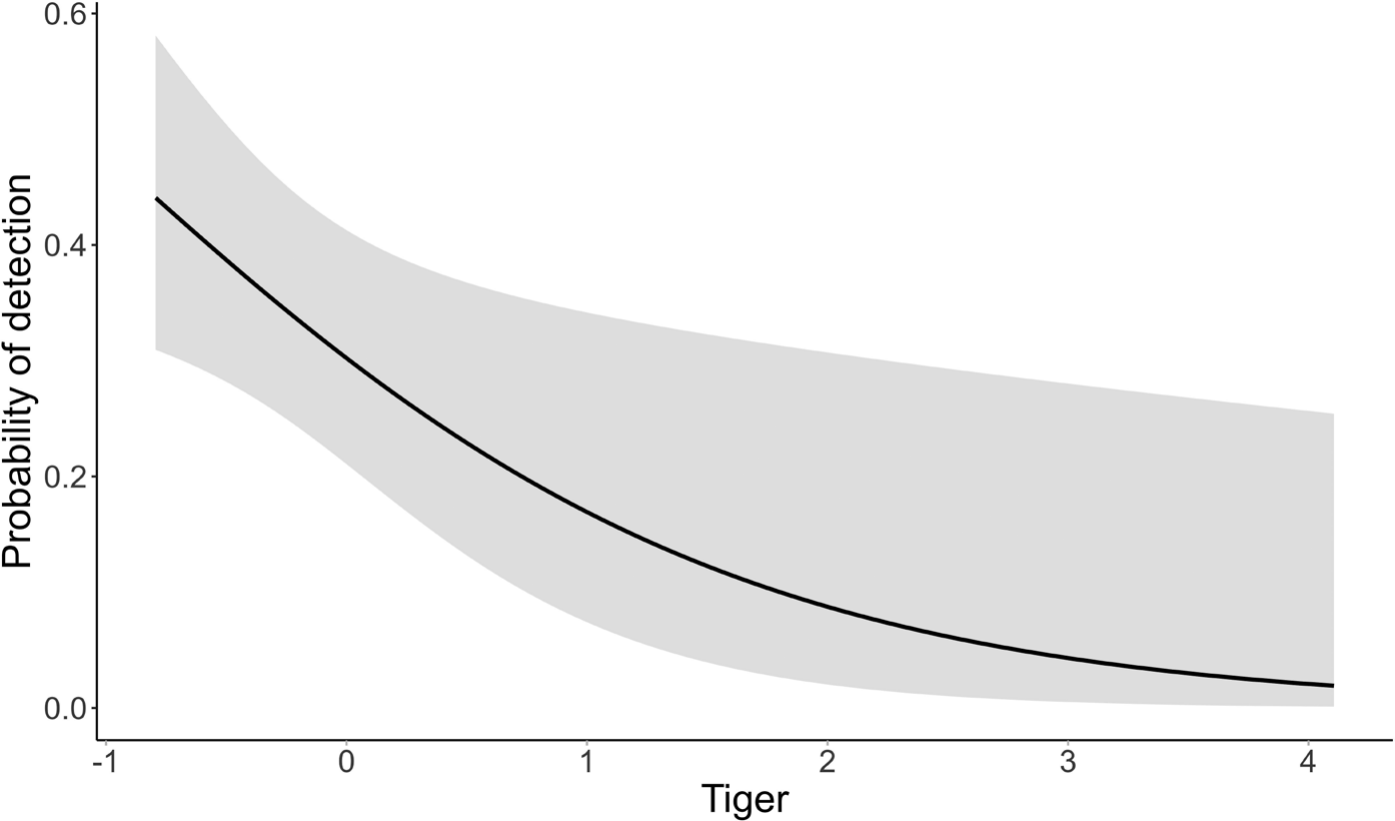
Relationship between tiger presence and detection probability of leopards in the study area.

Our top model for probability of detection (Ψ (.)*P*(covariates)) indicated that the presence of tiger was the most influential habitat covariate (Table 1), negatively influencing leopard habitat use. The probability of detecting a leopard declined with increasing number of tigers (Figure 2).

**TABLE 1:** Summary of the model‐selection procedures for factors influencing the effect of fine‐scale covariates on leopard detection probability.

Models	NPars	AIC	Delta	AICwt	CumltvWt
*ρ*(tiger)	3	365.73	0	0.5691	0.57
*ρ*(tiger + tri)	4	367.71	1.98	0.2117	0.78
*ρ*(river)	3	370.2	4.47	0.061	0.84
*ρ*(livestock + river)	4	370.26	4.53	0.0592	0.9
*ρ*(settlement)	3	371.89	6.16	0.0261	0.93
*ρ*(tri + river)	4	371.98	6.25	0.025	0.95
*ρ*(prey + river)	4	372.19	6.45	0.0226	0.97
*ρ*(settlement + tri)	4	373.72	7.99	0.0105	0.99
*ρ*(.)	2	375.24	9.51	0.0049	0.99
*ρ*(prey)	3	375.51	9.78	0.0043	0.99
*ρ*(tri)	3	376.97	11.24	0.0021	1
*ρ*(livestock)	3	377.1	11.37	0.0019	1
*ρ*(prey + tri)	4	377.5	11.77	0.0016	1

*Note:* ΔAIC is the difference in AIC values between each model and the model with the lowest AIC.

Abbreviations: AIC, Akaike's information criterion; CumltvWt, Cumulative Weight; k, degrees of freedom; livestock, livestock density; nPars, Number of Parameters; prey, prey density; river, distance to river; settlement, distance to human settlement; tiger, index of tiger detection; tri, terrain ruggedness index; ρ, probability of detection.

The model‐specific β‐coefficient value from the occupancy models for the presence of prey (*β* = 0.311 ± 0.129_SE_), livestock (*β* = 0.0479 ± 0.012_SE_) indicated a positive influence on leopard detection, whereas the negative β‐coefficients for distance to settlement (*β* = −0.399 ± 0.17_SE_), distance to river (*β* = −0.48 ± 0.193_SE_), terrain ruggedness index (*β* = −0.0872 ± 0.01_SE_), and presence of tiger (*β* = −0.754 ± 0.227_SE_) indicated negative associations with leopard detection (Table 2).

**TABLE 2:** Comparison of the relative strength of covariate influence on leopard occupancy and detection.

Covariates	Occupancy			Detection		
	β (SE)	LCI	UCI	β (SE)	LCI	UCI
Settlement	0.728 (0.125)	0.48	0.97	−0.399 (0.17)	−0.73	−0.07
River	0.6 (0.124)	0.36	0.84	−0.48 (0.193)	−0.86	−0.10
TRI	0.431 (0.101)	0.23	0.63	−0.0872 (0.01)	−0.11	−0.07
Tiger	0.762 (0.34)	0.10	1.43	−0.754 (0.227)	−1.20	−0.31
Prey	0.385 (0.002)	0.38	0.39	0.311 (0.129)	0.06	0.56
Livestock	0.489 (0.101)	0.29	0.69	0.0479 (0.012)	0.02	0.07

### 3.2 Probability of Occupancy

**FIGURE 3:**
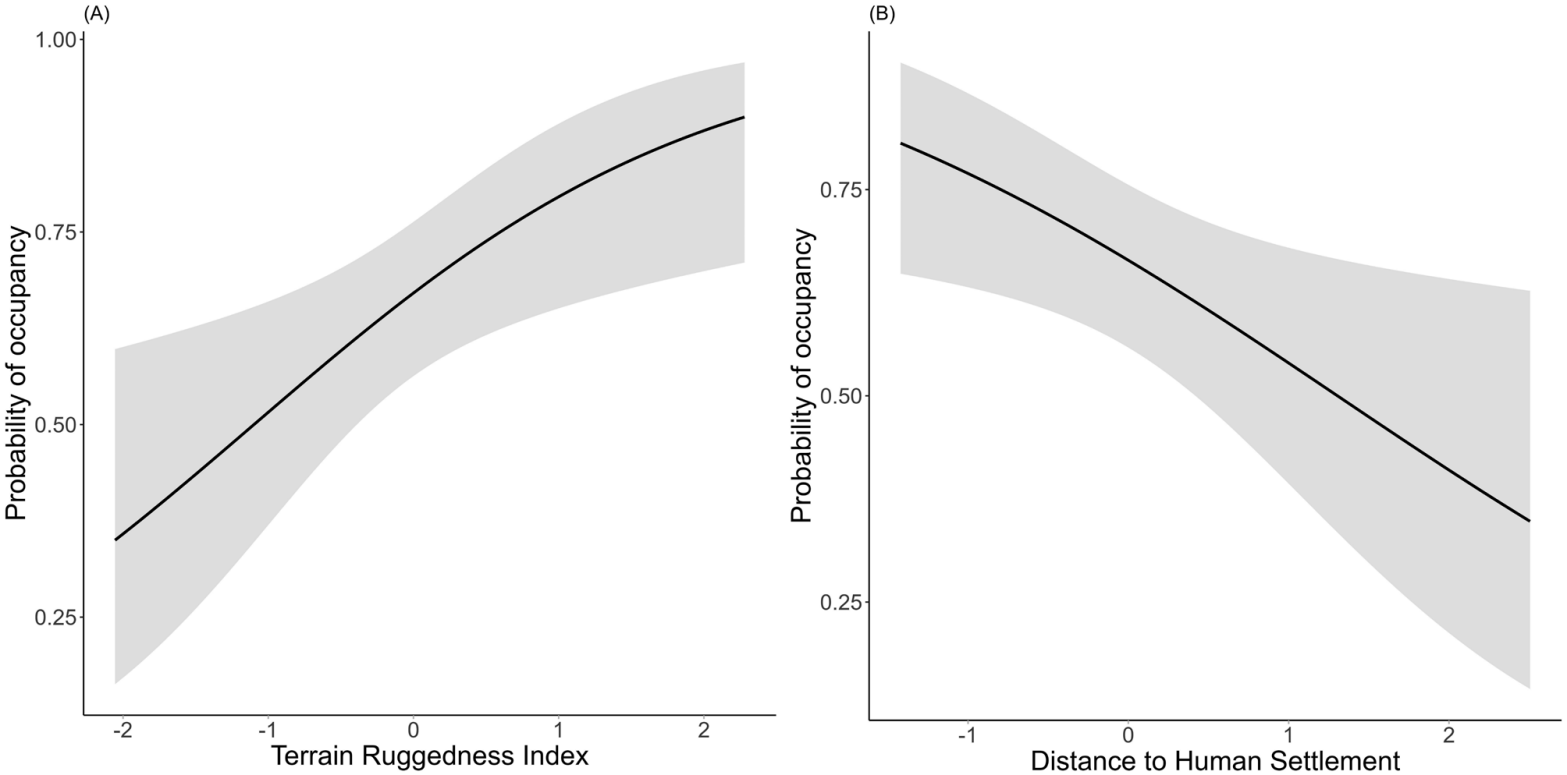
Relationship between distance to human settlement and probability of occupancy (right side) and terrain ruggedness index and probability of occupancy (left side).

We used the detectability models in subsequent analyses to model the occupancy probability (Table 3). Among the evaluated models, the model with distance to settlement and terrain ruggedness index “p(tiger) Ψ (settlement + tri)” had the highest rank (ΔAIC = 0) (Figure 3). The covariate tiger, settlement, and terrain ruggedness index together were valuable for estimating leopard occupancy.

**TABLE 3:** Summary of the model‐selection procedures for factors influencing leopard occupancy.

Models	NPars	AIC	Delta	AICwt	CumltvWt
ψ(settlement + tri) *p*(tiger)	5	358.2	0	0.6257	0.63
ψ(settlement + river) *p*(tiger)	5	362.11	3.91	0.0886	0.71
ψ(livestock + tri) *p*(tiger)	5	363.89	5.69	0.0364	0.75
ψ(settlement) *p*(tiger)	4	364.14	5.94	0.0322	0.78
ψ(tri) *p*(tiger)	4	364.16	5.96	0.0318	0.81
ψ(river + tri) *p*(tiger)	5	364.27	6.07	0.03	0.84
ψ(river + prey) *p*(tiger)	5	364.62	6.42	0.0253	0.87
ψ(river) *p*(tiger)	4	364.86	6.66	0.0224	0.89
ψ(livestock + river) *p*(tiger)	5	364.96	6.75	0.0214	0.91
ψ(settlement + prey) *p*(tiger)	5	365.23	7.03	0.0186	0.93
ψ(.) *p*(tiger)	3	365.73	7.53	0.0145	0.95
ψ(livestock + settlement) *p*(tiger)	5	365.94	7.74	0.0131	0.96
ψ(prey + tri) *p*(tiger)	5	365.96	7.76	0.0129	0.97
ψ(livestock) *p*(tiger)	4	366.56	8.36	0.0096	0.98
ψ(tiger) *p*(tiger)	4	366.63	8.43	0.0093	0.99
ψ(prey) *p*(tiger)	4	366.85	8.65	0.0083	1

## 4 Discussion

This study provides important insight into leopard habitat use in southwestern Tarai by identifying the covariates influencing occupancy and detection probability. We found a naïve occupancy of 0.51, and a model‐averaged occupancy estimate of 0.6563 (SE: 0.022, 95% CI: 0.612, 0.70), thus indicating that leopards were widely distributed across the park, especially in the fringe area (Appendix S2). A wide range of occupancy estimates were previously reported from various parts of Nepal. For example, Adhikari (2022) reported the highest estimated occupancy of 0.94 for leopards in the Chitwan Annapurna Landscape in central Nepal. Similarly, Lamichhane et al. (2021) reported a probability of leopard occupancy as 0.70 in the western Chure region and 0.46 in the eastern Chure region of Nepal. Thapa et al. (2021) documented a probability of leopard occupancy as 0.86 in the Terai Arc Landscape (TAL) in 2009, which later declined to 0.59 in 2013. The variation in the probability of leopard occupancy among the studies might be due to different sizes of survey area and grids, and various other factors, such as whether the studies were conducted within or outside protected areas, as well as their proximity to human settlements, and importantly the presence and absence of the tigers.

We found a negative influence of tiger presence on the detection probability of leopards. Leopards might have been pushed to the marginal areas of the park, possibly to minimize predation risk from tigers who prefer areas less disturbed by humans (Carter et al. 2015; Harihar, Pandav, and Goyal 2011; Kafley et al. 2019; Lamichhane et al. 2021; Steinmetz, Seuaturien, and Chutipong 2013; Thapa et al. 2021). Tigers have been observed pursuing and killing leopards (Kumar et al. 2019), and the leopards' use of marginal areas near settlements fits a pattern expected from interference competition with the increasing tiger population in the core area of the park (Odden, Wegge, and Fredriksen 2010; Rayan and Linkie 2016). Moreover, leopards may select marginal areas along the park border to access easily available domestic prey (Kshettry, Vaidyanathan, and Athreya 2018; Odden and Wegge 2005). Contrary to these findings, leopards are found to avoid humans in some parts of Asia (Palei et al. 2022), and exhibit temporal avoidance of humans by increasing nocturnality (Carter et al. 2015).

The covariates “settlement” and “terrain ruggedness index” were the most important factors influencing leopard occupancy. The highly adaptable leopard is a generalist species that is able to thrive in human‐modified landscapes (Acharya et al. 2017; Odden et al. 2014), near human habitations, and with moderate vegetation cover (Balme, Hunter, and Slotow 2007; Naha, Sathyakumar, and Rawat 2018). The positive influence of the terrain ruggedness may be associated with avoidance of direct interactions with tigers, and rugged terrain may provide cover for ambush attacks (Lamichhane et al. 2021; Poudel et al. 2023).

Although prey selection of tigers and leopards may overlap significantly (Harihar, Pandav, and Goyal 2011; Kafley et al. 2019), some studies report a higher flexibility in leopard diets, and often larger proportions of domestic livestock (Kandel, Lamichhane, and Subedi 2020; Lovari et al. 2015; Odden, Wegge, and Fredriksen 2010; Odden and Wegge 2005). In landscapes heavily modified by humans, leopards have been shown to be almost completely dependent on domestic animals as prey (Athreya et al. 2016). Correspondingly, Pokheral and Wegge (2018) also reported spatial segregation between leopards and tigers in ShNP. Their findings suggested that the segregation is not because of low predator–prey ratios; rather, both species' distribution patterns followed the abundance of their preferred prey (Pokheral and Wegge 2018). This suggests that some level of disturbances by humans and livestock may mediate the coexistence between leopards and tigers in small, protected areas like ShNP (Kafley et al. 2019; Lamichhane et al. 2019). The covariates “settlement” and “livestock” are related to each other. Specifically, the buffer zone of ShNP is used by livestock‐dependent communities, and livestock freely range in the fringe areas of the park.

Nepal has recently reached a milestone in tiger conservation (DNPWC and DFSC 2022). Conservation efforts in Nepal's Tarai are mostly centered on some umbrella species, including the tigers, whereas leopards are neglected. It is expected that the increasing tiger population will further push leopards to the marginal areas, inducing more conflicts with communities, and thereby threaten the persistence of leopards (Baral et al. 2022). Thus, a proper understanding of the ecological interactions between leopards and tigers is required to ensure sustainable management of both species.

## 5 Conclusion

Understanding the habitat use of leopards and their ability to coexist with tigers is vital for the development of sustainable conservation and management strategies. The results of this study have important implications for leopard conservation in Nepal's Tarai landscape. At a time when ShNP and other protected areas in Nepal's Tarai have achieved their tiger conservation targets, intensive efforts are required to maintain adequate prey biomass in the park area to minimize fatal encounters between tigers and leopards and further displacement of leopards into fringe areas where they might be killed in retaliation. Our findings are not only relevant to Nepal but also important for countries where leopards and tigers coexist, contributing significantly to the assessment of ecological influencing factors and their sustainable conservation. Long‐term monitoring is required to understand the interaction between leopards, tigers, and humans living near the periphery of the park. In future, it is also important to examine multi‐season and multispecies occupancy to better understand their interactions and to inform larger carnivore conservation strategies in Nepal.

## Author Contributions

**Laxmi Raj Joshi:** conceptualization (lead), data curation (lead), formal analysis (lead), methodology (lead), writing – original draft (lead), writing – review and editing (lead). **Rabin Bahadur K. C.:** conceptualization (equal), data curation (equal), formal analysis (equal), investigation (lead), methodology (equal), writing – original draft (equal), writing – review and editing (equal). **Madhu Chetri:** conceptualization (equal), methodology (equal), supervision (lead), writing – review and editing (equal). **Morten Odden:** conceptualization (equal), methodology (equal), supervision (equal), writing – review and editing (equal). **Olivier Devineau:** methodology (equal), supervision (equal), writing – review and editing (equal). **Ajay Karki:** supervision (equal), validation (equal), writing – review and editing (equal). **Bhagawan Raj Dahal:** supervision (equal), writing – review and editing (equal). **Naresh Subedi:** conceptualization (equal), funding acquisition (lead), methodology (equal), supervision (equal), validation (equal), writing – review and editing (equal).

## Conflicts of Interest

The authors declare no conflicts of interest.

## Supporting information


Appendix S1.

Appendix S2.

## Data Availability

Data associated with this manuscript can be accessed at the Dryad data repository (http://datadryad.org/stash/share/‐k30AV9BqFk6zIHiYdAulUblUr4qAKNGgPqjFNr0rqY).
