# Supplementary material for: Tigers, Terrain, and Human Settlement Influence the Occupancy of Leopards (Panthera pardus) in Southwestern Tarai, Nepal
Source: Ecol Evol. 2025 Jan 20;15(1):e70898. doi: 10.1002/ece3.70898 (PMC11745899; doi:10.1002/ece3.70898)
Supplement: Supplementary file 1 — Appendix S1. [file ECE3-15-e70898-s001.docx]

**Tigers, Terrain, and Human Settlement Influence the Occupancy of Leopards (*Panthera pardus*) in Southwestern Tarai, Nepal**

Laxmi Raj Joshi^1*^, Rabin Bahadur K.C. ^1^, Madhu Chetri^1^, Morten Odden^2^, Olivier Devineau^2^, Ajay Karki^3^, Bhagawan Raj Dahal^4^, Naresh Subedi^1^

^1^National Trust for Nature Conservation, P.O. Box. 3712, Khumaltar, Lalitpur, Nepal. ^2^Faculty of Applied Ecology, Agricultural Sciences and Biotechnology, Inland Norway University of Applied Sciences, NO-2480 Koppang, Norway.

^3^Department of National Parks and Wildlife Conservation, Babar Mahal, Kathmandu, Nepal.

^4^Zoological Society of London Nepal Office, Kathmandu, Nepal.

Email Address of Authors:

Rabin Bahadur K.C., Email: [rbn.kc80@yahoo.com](mailto:rbn.kc80@yahoo.com)

Madhu Chetri, Email: [mchetri@gmail.com](mailto:mchetri@gmail.com)

Morten Odden, Email: [morten.odden@inn.no](mailto:morten.odden@inn.no)

Olivier Devineau, Email: [olivier.devineau@inn.no](mailto:olivier.devineau@inn.no)

Ajay Karki, Email: [clickajaya@gmail.com](mailto:clickajaya@gmail.com)

Bhagwan Raj Dahal, Email: [BhagawanRaj.Dahal@zsl.org](mailto:BhagawanRaj.Dahal@zsl.org)

Naresh Subedi, Email: [nareshsubedi@gmail.com](mailto:nareshsubedi@gmail.com)

**^*^Corresponding author:** Laxmi Raj Joshi; Email: [lrjoshi@ntnc.org.np](mailto:lrjoshi@ntnc.org.np)

**Supplementary file Table S1: Description of the covariate’s sources and the description regarding the use of the covariates in the hypothesis.**

| **Groups** | **Covariates** | **Source details** | **Description** |
| --- | --- | --- | --- |
| Topographical Variables | Topographic Ruggedness Index | Extracted from Digital Elevation Model (DEM) downloaded from Worldclim.org (https://www.worldclim.org/) in ArcGIS | Churia is hilly region where higher elevations can also be especially rugged. Tigers and their prey should tend to avoid area with high TRI. |
| Ecological Variables | Distance to river | <https://rds.icimod.org/home/datadetail?metadataid=852> | The detection of the leopards are highly influencing factors, as they are mostly dependent to the river for water as well as for the prey (Khodri et al., 2021) |
|  | Distance to Road | <https://rds.icimod.org/home/datadetail?metadataid=3620> | Linear infrastructure development, such as construction of roads and canals, is an emerging threat that triggers large scale habitat fragmentation (Trombulak & Frissell, 2000). Road networks can fragment large areas of continuous habitat into smaller patches (Saunders et al., 2002; Mutter et al., 2015). Road abundance for larger roads in potential habitat will negatively influence leopard presence across the landscape. However, small cart roads will positively influence occupancy and detection of leopard sign along the potential habitat within in grid cells as leopards can readily use such small dirt roads for travel. |
|  | Tiger | Field Survey (Camera Trapping) | It is expected tiger could suppressed other sympatric carnivores. The RAI value of oTiger captured in each grid were calculated. |
|  | Prey Activity Index | Field Survey (Camera Trapping) | Carnivore like tiger densities are known to be a function of prey biomass and density (Karanth et al., 2011). Thus, leopard presence should be governed by the availability of wild prey. |
|  | Livestock detection | Field Survey (Camera Trapping) | Leopards are highly influenced by the presence of domestic livestock. |
|  | Distance to Nearest Settlement | [OpenStreetMap](https://www.openstreetmap.org/#map=4/26.16/98.26) | Settlement used as a surrogate measure of disturbance at the landscape level and is an easily quantifiable measure that correlates with human activity (Gray & Phan, 2011). |
